# FEDRR: fast, exhaustive detection of redundant hierarchical relations for quality improvement of large biomedical ontologies

**DOI:** 10.1186/s13040-016-0110-8

**Published:** 2016-10-10

**Authors:** Guangming Xing, Guo-Qiang Zhang, Licong Cui

**Affiliations:** 1 Department of Computer Science, Western Kentucky University, Bowling Green, 42101 KY USA; 2 Institute of Biomedical Informatics, University of Kentucky, Lexington, 40536 KY USA; 3 Department of Computer Science, University of Kentucky, Lexington, 40506 KY USA

**Keywords:** Redundant relations, Dynamic programming, SNOMED CT, Gene ontology, UMLS

## Abstract

**Background:**

Redundant hierarchical relations refer to such patterns as two paths from one concept to another, one with length one (direct) and the other with length greater than one (indirect). Each redundant relation represents a possibly unintended defect that needs to be corrected in the ontology quality assurance process. Detecting and eliminating redundant relations would help improve the results of all methods relying on the relevant ontological systems as knowledge source, such as the computation of semantic distance between concepts and for ontology matching and alignment.

**Results:**

This paper introduces a novel and scalable approach, called FEDRR – Fast, Exhaustive Detection of Redundant Relations – for quality assurance work during ontological evolution. FEDRR combines the algorithm ideas of Dynamic Programming with Topological Sort, for exhaustive mining of all redundant hierarchical relations in ontological hierarchies, in *O*(*c*·|*V*|+|*E*|) time, where |*V*| is the number of concepts, |*E*| is the number of the relations, and *c* is a constant in practice. Using FEDRR, we performed exhaustive search of all redundant is-a relations in two of the largest ontological systems in biomedicine: SNOMED CT and Gene Ontology (GO). 372 and 1609 redundant is-a relations were found in the 2015-09-01 version of SNOMED CT and 2015-05-01 version of GO, respectively. We have also performed FEDRR on over 190 source vocabularies in the UMLS - a large integrated repository of biomedical ontologies, and identified six sources containing redundant is-a relations. Randomly generated ontologies have also been used to further validate the efficiency of FEDRR.

**Conclusions:**

FEDRR provides a generally applicable, effective tool for systematic detecting redundant relations in large ontological systems for quality improvement.

## Background

Ontologies are shared conceptualizations of a domain represented in a formal language. They represent not only the concepts (nodes) but the relationships (edges) between the concepts. Ontologies have become a critical knowledge source in informatics and data intensive applications, such as information retrieval [1], data integration [2], data management [3], and decision support [4].

This paper focuses on a particular type of ontological structural defect: redundant relations. Redundant hierarchical relations refer to such patterns as two paths from concept *X* to concept *Y*, one with length one (direct) and the other with length greater than one (indirect). For hierarchical relations such as subsumption (is-a), relations implied by transitivity should not be explicitly stated. For example, in Gene Ontology (GO 2015-05-01 version) we have (see Table 1). A (hormone secretion) is-a B (hormone transport), B (hormone transport) is-a C (regulation of hormone levels), C (regulation of hormone levels) is-a D (regulation of biological quality), D (regulation of biological quality) is-a E (biological regulation), E (biological regulation) is-a F (biological process).

**Table 1:** Indirect path from concept A (hormone secretion) to concept F (biological process) in GO (2015-05-01 version)

	GO Id	Relation		GO Id
A	GO:0046879	is-a	B	GO:0009914
B	GO:0009914	is-a	C	GO:0010817
C	GO:0010817	is-a	D	GO:0065008
D	GO:0065008	is-a	E	GO:0065007
E	GO:0065007	is-a	F	GO:0008150

**Fig. 1:**
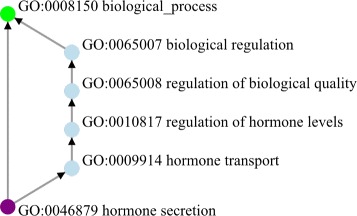
Graphical rendering of Table 1 and a direct edge between A and F. Directed edges represent “is-a” relation

However, “A (GO:0046879) is-a F (GO:0008150)” is directly asserted as well (Fig. 1). This represents redundant relations to be studied in this paper: two paths exist between A and F: one directly between A and F, and the other indirectly through B, C, D, and E as intermediate concept nodes.

The principle of parsimony in ontological modeling refers to the omission of relations implied by the transitive property of a relationship, such as “is-a” relations in GO. By violating this principle, redundant relations may increase maintenance burden for ontology curators. It can also cause inaccurate methods and algorithms based on this general principle. For example, semantic distance between concepts is a widely used technique [5]. Ontological mapping and alignment methods rely on the ordered structure of the hierarchical relation [6], with notions of neighborhood and proximity serving as their foundation. The presence of redundant relations induces a short-circuit: two concepts with a larger semantic distance may result in a smaller distance by mistake; and concepts not within a neighborhood may be counted as such.

Using brute force, exhaustive detection of redundant relations can be computationally expensive for large ontologies. For example, SNOMED CT (2015-03-01 version) contains over 300,000 active concepts. A naive approach would be to find the longest paths between the end nodes of each of the over 500,000 edges (relations). *Assuming each edge takes 100 ms, processing a single version of SNOMED CT would take 14 hours.* Finding all paths between all possible pairs among the 300 k nodes would take over 10,000 days if each pair takes 10 ms.

This paper introduces a novel and scalable approach, called FEDRR, Fast, Exhaustive Detection of Redundant Relations, for quality assurance work during ontological evolution. In contrast to the 14 hours naive approach required for each SNOMED CT version, *FEDRR needed <20 seconds* (see Table 2).

**Table 2:** Summary of the results for 5 versions of SNOMED CT

Version	# Concepts	# is-a Relations	TC	RR	RR %	T(ms)
2013-09-01	300,485	447,442	5,226,630	240	0.00459	10,472
2014-03-01	300,409	446,603	5,188,221	277	0.00534	10,335
2014-09-01	302,902	449,564	5,222,506	305	0.00584	10,074
2015-03-01	315,904	467,799	5,408,010	235	0.00435	15,264
2015-09-01	320,911	476,226	5,511,334	372	0.00675	16,077

TC: number of transitive closure pairs, RR: number of redundant is-a relations, T(ms): time taken in milliseconds

Using FEDRR, we performed exhaustive search of all redundant is-a relations in two of the largest ontological systems in biomedicine: SNOMED CT and GO. 372 and 1609 redundant is-a relations were found in the SNOMED CT (2015-09-01 version) and GO (2015-05-01 version), respectively. Each redundant relation represents a possibly unintended defect that needs to be corrected in the ontology quality assurance process. We further performed longitudinal analyses using FEDRR on 5 versions of SNOMED CT and 10 versions of GO. We also investigated redundant is-a relations in the UMLS, an integrated repository of biomedical terminologies, including SNOMED CT and GO.

### SNOMED CT

SNOMED CT is the world’s largest clinical terminology [7, 8]. It provides broad coverage of clinical medicine, including findings, diseases, and procedures for use in electronic medical records.

From a structural perspective, SNOMED CT can be seen as a series of large directed acyclic graphs, one for each of its 19 “sub-hierarchies” including Procedure, Substance, Body structure, Specimen, Clinical finding, and Organism. No concept is shared across sub-hierarchies except for the root. Each concept comes with a SNOMED CT identifier, which is an integer. SNOMED CT concepts are linked by hierarchical relations within each sub-hierarchy.

### Gene ontology

The Gene Ontology (GO) [9] is a collection of three ontologies to describe attributes of gene products in three non-overlapping domains of molecular biology: Cellular Component, the parts of a cell or its extracellular environment; Molecular Function, the elemental activities of a gene product at the molecular level, such as binding or catalysis; and Biological Process, operations or sets of molecular events with a defined beginning and end, pertinent to the functioning of integrated living units (cells, tissues, organs, and organisms). Within each ontology, terms have free text definitions and unique identifiers. GO terms can be related to each other by is-a and part-of relationships, forming a directed acyclic graph. The GO vocabulary is designed to be species-agnostic, and is intended to capture multiple organisms.

### UMLS

The Unified Medical Language System (UMLS) [8], produced and distributed by US National Library of Medicine (NLM), is a large integrated repository of biomedical controlled vocabularies to facilitate interoperability among disparate systems in biomedicine and health. The source vocabularies include SNOMEDCT_US (SNOMED CT US Edition), SCTSPA (SNOMED CT Spanish Language Edition), GO, FMA (Foundational Model of Anatomy), HPO (Human Phenotype Ontology), and NCI (NCI Thesaurus).

Knowledge in the UMLS Metathesaurus is organized by concept (or meaning). Term variants from source vocabularies are clustered together to form a concept, and each concept is assigned a unique concept identifier (CUI). The basic building blocks (or atoms) of the UMLS Metathesaurus are the concept names or strings from each source vocabulary. Every occurrence of a string in each source is assigned a unique atom identifier (AUI). For instance, concept names *Hypertension*, *High blood pressure*, and *Hypertensive disorder* from SNOMEDCT_US have AUIs A2882711, A2876587, and A3501627, respectively. *Hypertension* and *Vascular Hypertensive Disorder* from NCI has AUIs A7571194 and A7628940, respectively. Such concept names from different sources represent the same meaning and are assigned a unique CUI: C0020538. Moreover, relationships between terms in source vocabularies are preserved in the UMLS as relationship attributes.

The 2015AB release of the UMLS contains over 3.2 million concepts and 12.8 million unique concept names from more than 190 source vocabularies.

### Ontology quality assurance

Large, comprehensive terminological systems such as SNOMED CT and GO continue to evolve over time. They are often incomplete, under-specified, and non-static, for reasons such as the evolving state of knowledge in a domain, the involvement of manual curation work, and the progressive nature of ontological engineering itself. New applications are calling for new ontologies or expansion and enhancement of existing ones. Many additional factors, such as merging or reusing existing ontologies and porting to a common representation framework, may introduce inconsistencies and unintended artifacts. Thus Ontology Quality Assurance (OQA) is an indispensable part of the ontological engineering lifecycle [10–21]. OQA attempts to assess and improve the overall quality of ontologies in aspects such as the consistency of the ontological structure with respect to the explicit and implicit knowledge they capture; the coverage of the ontology in terms of classes and properties needed to support specific applications; and the non-redundancy of classes and properties.

The basic premise of OQA is a mixed closed-world assumption (CWA) and open-world assumption (OWA). In a formal system of logic used for knowledge representation, such as ontological systems, CWA refers to the assumption that a relationship holds true between two concepts is also explicitly asserted to be true, unless they are implied by logical properties such as transitivity. It dictates that, in reverse, a relationship between two concepts that is not asserted explicitly, must be false. OWA, on the other hand, refers to the assumption that lack of knowledge does not imply falsity.

In the context of OQA, OWA refers to the evolving state of knowledge in a domain, in the sense that new concepts may be included in an ontological system in a continuous fashion. The lack of a concept in an ontological system does not imply that such a concept does not exist. CWA, on the other hand, implies that, among existing concepts in an ontological system, the lack of an explicit relationship of a known relation-type between two concepts means that such a relationship does not exist between the two concepts.

The *principle of parsimony* in ontological modeling is a direct consequence of CWA. It refers to the fact that relations implied by the transitive property of a relationship, such as the example given in Fig. 1, must not be explicitly stated. By violating this principle, redundant relations can cause methods and algorithms based on this general principle inaccurate. Detecting redundant relations is an important task for OQA, which is the focus of this paper.

## Methods

The general mathematical abstraction of an ontological structure is a graph-theoretic one: nodes correspond to concepts, and edges correspond to relations (between nodes). For hierarchical relations in ontological systems such as “is-a,” which obeys the *transitivity property* that

$$\begin{array}{@{}rcl@{}} \text{if } A \text{ is-a } B \text{ and } B \text{ is-a } C, \text{ then } A \text{ is-a } C, \end{array} $$

one can model the structure of an ontological system as a directed acyclic graph (DAG, as shown in part in Fig. 1).

### Definition 1.

Suppose *G*=(*V*,*E*) is a directed acyclic graph with *V* a set of nodes, and *E* a set of edges between the nodes. A redundant relation in *G* is a pair of nodes (*s*,*t*) such that (*s*,*t*)∈*E*, and there is an indirect path (i.e., length more than 1) from *s* to *t*.

The closely related known algorithm for computing redundant relations in the literature is all-pair longest path [22]. Although fixed source longest path can be solved in time-complexity *O*(|*V*|+|*E*|) in a DAG [22], all-pair longest path requires iteration over *V*, resulting in an *O*(|*V*|·|*E*|+|*V*|^2^) time-complexity algorithm. For large ontological systems such as SNOMED CT, such a running time amounts to an intractable amount of processing time (requiring 10,000 days if all-pair paths were to be computed).

FEDRR solves this problem in time-complexity *O*(*c*·|*V*|+|*E*|), where *c* is the average number of descendants of a node. For the latest version of SNOMED CT, we have *c*=17.12 (see Time Complexity Analysis). For a single version of SNOMED CT, the actual processing time is less than *20 seconds.*

There are two key algorithmic ideas behind FEDRR. One is avoidance of repeated computations by remembering the set of directly reachable nodes as well as the set of indirectly reachable nodes, for each node. The second is to completely skip node pairs that are not connected by a directed path. These ideas are reflected in FEDRR using a novel combination of dynamic programming with topological sort. The sparsity of most ontological structures, viewed as a DAG, is a particularly suitable property for the second idea to take advantage of.

For a node *u* in a DAG *G*=(*V*,*E*), we introduce two sets, *D*_*u*_ and *I*_*u*_, where

- *D*_*u*_={*v*∣(*v*,*u*)∈*E*}, called the *D*-**set**, consists of the direct descendants (i.e. children) of *u*; and
- *I*_*u*_, called the *I*-**set** of *u*, is the set of all indirect descendants of *u*.

The design of our algorithm is based on the following observation.

### Lemma 1.

For each node *v*∈*D*_*u*_∩*I*_*u*_, (*v*,*u*) is redundant.

Our algorithm amounts to the computation of (*D*_*u*_,*I*_*u*_) for each node *u*. To utilize the idea of dynamic programming, we update (*D*_*u*_,*I*_*u*_) for each node *u* according to the order by topological sort. The basic update scheme is illustrated in the following diagram:

Suppose we have obtained $(D_{v_{i}}, I_{v_{i}})$ for each *i*=1,…,*k*, where {*v*_1_,*v*_2_,…}={*v*∣(*v*,*u*)∈*E*}. Then we set *D*_*u*_={*v*_1_,*v*_2_,…} and $I_{u} = \bigcup _{i=1}^{k} (D_{i} \cup I_{i}).$ The pseudo-code of FEDRR appears in Algorithm 1.



**Fig. 2:**
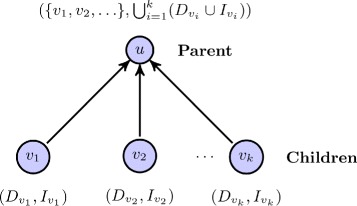
Basic mechanism for updating the *D*-set and the *I*-set of a node

FEDRR starts by initializing an empty queue to hold the nodes that will be sorted (line 2). Then nodes with no incoming edges are put to the queue, with the *D*-set and *I*-set initialized as empty (lines 3 - 9). In the next phase (lines 10 - 20), the nodes are dequeued one at a time, with the *I*-sets and *D*-sets (for *t*) updated according to the mechanism described in Fig. 2.

**Fig. 3:**
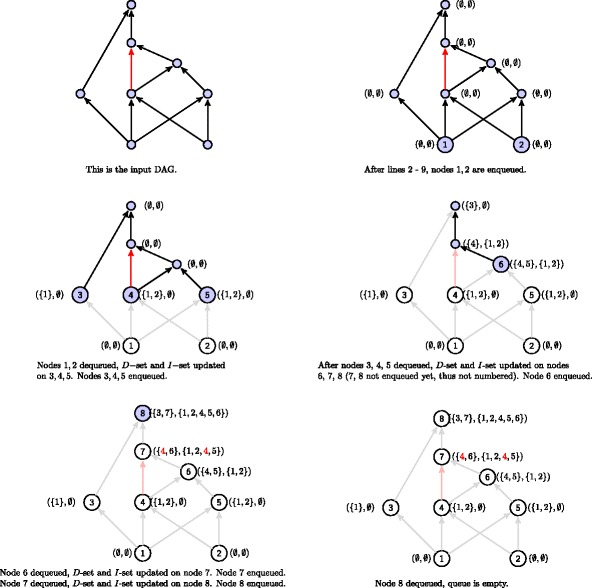
Illustration of Algorithm 1

We illustrate the steps of Algorithm 1 using an example. The input DAG is given in Fig. 3 (top left), and there is a redundant edge (colored in red) that FEDRR is supposed to detect. The algorithm starts with setting initial values for the *D*-set and the *I*-set and enqueuing those node with no incoming edges, as shown on the top right of Fig. 3. The result is shown on the bottom right of Fig. 3.

### Correctness

The correctness of the algorithm can be proved using mathematical induction by showing $\phantom {\dot {i}\!}I[v_{i}] = I_{v_{i}}$ and $\phantom {\dot {i}\!}D[v_{i}]=D_{v_{i}}$ after node *v*_*i*_ is dequeued (line 11) for *i*=1…|*V*|.

#### *Proof*

*i*=1. The first dequeued node must be a node with no incoming edges. This means $\phantom {\dot {i}\!}I_{v_{1}} =\emptyset $ and $D_{v_{1}}=\emptyset $. As both *I*[*v*_1_]=*∅* and *D*[*v*_1_]=*∅* from lines 4 and 5, we have $\phantom {\dot {i}\!}I[v_{1}] = I_{v_{1}}$ and $\phantom {\dot {i}\!}D[v_{1}] = D_{v_{1}}$.

Suppose $\phantom {\dot {i}\!}I[v_{i}] = I_{v_{i}}$ and $\phantom {\dot {i}\!}D[v_{i}] = D_{v_{i}}$ is true for *i*=1…*k*−1. For *i*=*k*, then we have *D*[*v*_*k*_]={*v*∣(*v*,*v*_*k*_)∈*E*} and $\phantom {\dot {i}\!}I[v_{k}] = \bigcup _{j} (D[v_{k_{j}}] \cup I[v_{k_{j}}]),$ where $v_{k_{j}}\in \{ v \mid (v, v_{k}) \in E\} $. Based on the definition of *D*_*v*_, we have $D_{v_{k}} = \{ v \mid (v, v_{k}) \in E\} = D[v_{k}] $. From the induction hypothesis, we have $\phantom {\dot {i}\!}I[v_{i}] = I_{v_{i}}$ and $\phantom {\dot {i}\!}D[v_{i}] = D_{v_{i}}$ for *i*=1…*k*−1. This means $I[v_{k}] = \bigcup _{j} \left (D[v_{k_{j}}] \cup I[v_{k_{j}}]\right) = \bigcup _{j} \left (D_{v_{k_{j}}} \cup I_{v_{k_{j}}}\right) = I_{v_{k}}.\phantom {\dot {i}\!}$ □

### Time complexity analysis

The topological sorting itself takes *O*(|*V*|+|*E*|) time [23]. With the computation of *D*-set and *I*-set, the total time is $O(\sum _{(u,v)\in E}(|D_{v}|+ |I_{v}|) + |V|+|E|)$. When |*E*|=*O*(|*V*|) (which is the case for both SNOMED CT and GO), the running time is $O(\sum _{v}(|D_{v}|+ |I_{v}|) + |V|+|E|)$. If we let $c=\frac {\sum _{v}(|D_{v}|+|I_{v}|)}{|V|}$, then the running time is in *O*(*c*·|*V*|+|*E*|). Based on the definition of *D*_*v*_ and *I*_*v*_, $\sum _{v}(|D_{v}|+|I_{v}|)$ is the size of transitive closure pairs shown in Tables 2 and 4. Even though the worst-case running time is *O*(|*V*|^2^)(when *c*=|*V*|), *c* is a relatively small constant for ontological systems in practice. This is validated by our experimental results shown in Tables 2 and 4. For the 2015-03-01 version of SNOMED CT, $c=\frac {5,408,010}{315,904}=17.12$, and for the 2015-05-01 version of GO, $c=\frac {557,550}{42,979}=12.97$.

## Results

### Experimental environment

To detect redundant is-a relations from SNOMED CT and Gene Ontology, we ran the FEDRR method on a MacBook Pro running the Mac OS X Yosemite with 16 GB RAM and Intel Core i7 processor. FEDRR was implemented in Java programming language based on JDK7.

### Redundant is-a relations in SNOMED CT

We ran the FEDRR method on 5 versions of SNOMED CT (U.S. edition) from 2013 to 2015 dated on 2013-09-01, 2014-03-01, 2014-09-01, 2015-03-01, and 2015-09-01. Table 2 summarizes the result of each version including numbers of concepts, is-a relations, and transitive closure pairs (TC), and number of redundant is-a relations (RR); percentage of redundant is-a relations (RR%) among transitive closure pairs; and computing time in milliseconds to detect redundant is-a relations. For example, for the 2015-09-01 version, there were 320,911 concepts, 476,226 is-a relations, 5,511,334 transitive closure pairs, and 372 redundant is-a relations; the percentage of the redundant is-a relations among the transitive closure pairs is 0.00675 %; and it took about 16 seconds to complete. For each version, it only took a few seconds to identify all the redundant is-a relations, indicating the efficiency of FEDRR.

Table 3 shows the numbers of redundant is-a relations in 5 versions of SNOMED CT with respect to the length of the indirect path. For each version, *l*_*i*_(*i*=2,3,4) is the number of redundant is-a relations in length of *i* regarding to the indirect path. For example, in the version of 2015-09-01, there were 358 redundant is-a relations in length of 2, 13 in length of 3, and 1 in length of 4. In general, most redundant is-a relations were in length of 2, and no redundant is-a relations exceeding length of 4 was identified.

**Table 3:** Numbers of redundant is-a relations in 5 versions of SNOMED CT regarding to the length of the indirect path. *l*_*i*_ represents the number of redundant is-a relations in length of *i* regarding to the indirect path

Version	*l*_2_	*l*_3_	*l*_4_	Total
2013-09-01	233	7	0	240
2014-03-01	264	11	2	277
2014-09-01	291	13	1	305
2015-03-01	224	10	1	235
2015-09-01	358	13	1	372

**Table 4:** Summary of the results for 10 versions of Gene Ontology

Version	# Concepts	# is-a Relations	TC	RR	RR%	T(ms)
2014-08-01	41,436	66,544	517,092	497	0.0961	1,372
2014-09-01	41,694	66,995	522,741	502	0.0960	1,472
2014-10-01	41,867	67,536	528,821	631	0.1193	1,455
2014-11-01	42,012	69,300	541,718	1,031	0.1903	1,497
2014-12-01	42,189	69,887	545,168	1,193	0.2188	1,425
2015-01-01	42,329	70,272	544,210	1,277	0.2347	1,510
2015-02-01	42,466	70,724	546,158	1,420	0.2600	1,549
2015-03-01	42,588	71,032	548,006	1,463	0.2670	1,542
2015-04-01	42,805	71,549	552,367	1,552	0.2810	1,437
2015-05-01	42,979	71,954	557,550	1,609	0.2886	1,538

TC: number of transitive closure pairs, RR: number of redundant is-a relations, RR%: percentage of redundant is-a relations among transitive closure pairs, T(ms): time taken in milliseconds

### Redundant is-a relations in gene ontology

We ran the FEDRR method to detect redundant is-a relations in 10 versions of Gene Ontology from 2014-08-01 to 2015-05-01 updated monthly. Table 4 summarizes the basic results of each version. For instance, for the 2015-05-01 version, there were 42,979 concepts, 71,954 is-a relations, 557,550 transitive closure pairs, and 1,609 redundant is-a relations; the percentage of the redundant is-a relations among the transitive closure pairs is 0.2886 %; and it took 1,538 milliseconds to complete. As the number of concepts and is-a relations were increasing, the number and percentage of redundant is-a relations (RR) were monotonically increasing every month and increased more than twice from the 2014-08-01 version (497; 0.0961 %) to the 2015-05-01 version (1,609; 0.2886 %). For each version, it only took a couple of seconds to identify all the redundant is-a relations, indicating the efficiency of FEDRR.

Table 5 shows the numbers of identified redundant is-a relations for the 10 versions with respect to the length of the indirect path. For each version, *l*_*i*_ (*i*=2,…,7) is the number of redundant is-a relations in length of *i* regarding to the indirect path. For example, in the version of 2015-05-01, there were 1,238 redundant is-a relations in length of 2 and 255 in length of 3. Most redundant is-a relations were in length of 2 or 3 regarding to the indirect path. There were only a couple of redundant is-a relations in length of 7. No redundant is-a relations exceeding length of 7 was identified.

**Table 5:** Numbers of redundant is-a relations in 10 different versions of Gene Ontology regarding to the length of the indirect path. *l*_*i*_ represents the number of redundant is-a relations in length of *i* regarding to the indirect path

Version	*l*_2_	*l*_3_	*l*_4_	*l*_5_	*l*_6_	*l*_7_	Total
2014-08-01	421	40	23	11	1	1	497
2014-09-01	419	44	24	13	1	1	502
2014-10-01	512	72	29	15	2	1	631
2014-11-01	771	164	64	27	4	1	1,031
2014-12-01	921	174	63	27	7	1	1,193
2015-01-01	980	202	62	24	8	1	1,277
2015-02-01	1,098	220	68	24	8	2	1,420
2015-03-01	1,119	237	72	25	8	2	1,463
2015-04-01	1,198	238	78	29	7	2	1,552
2015-05-01	1,238	255	78	29	7	2	1,609

### Redundant is-a relations in UMLS

We ran the FEDRR method to detect redundant is-a relations from over 190 source vocabularies integrated in the UMLS (2015AB release). Since the original occurrences of concept names are preserved and identified as AUIs in the UMLS, we used AUIs and relations between AUIs to detect redundant is-a relations. We also filtered out inactive is-a relations in the UMLS before applying the FEDRR method (obsolete relations are indicated by a value of ‘O’ in the SUPPRESS field in the relation file MRREL.RRF).

Based on our experiment, the concept graph in terms of AUI is acyclic, and FEDRR completes the exhaustive search for all the sources in our experiments. Six sources were found to have redundant is-a relations (see Table 6): SNOMEDCT_US (2015_09_01), SNOMEDCT_VET (2015_04_01), GO (2015_04_04), NCI (1502D), HPO (2015_04_20), and UMD (2015AA). For instance, in HPO, there were 18,175 AUIs, 14,762 is-a relations, 117,366 transitive closure pairs, and 101 redundant is-a relations. Moreover, it took FEDRR less than 30 seconds to detect redundant is-a relations for each of these source vocabularies.

**Table 6:** Summary of the results for source vocabularies in UMLS (2015AB release) with redundant relations

Version	# AUIs	# is-a Relations	TC	RR	RR%	T(ms)
SNOMEDCT_US	846,444	476,055	5,511,334	372	0.00675	28,977
SNOMEDCT_VET	85,939	19,832	29,688	7	0.024	954
GO	148,900	71,687	554,859	1,576	0.2840	9,128
NCI	270,618	119,707	701,986	20	0.0028	5,881
HPO	18,175	14,762	117,366	101	0.0861	502
UMD	34,124	10,750	37,732	20	0.0530	1,906

AUI: Atom Unique Identifier, TC: number of transitive closure pairs, RR: number of redundant is-a relations, T(ms): time taken in milliseconds

As can be seen from Table 6, SNOMEDCT_US in the UMLS shows the same number (372) of redundant is-a relations compared to the result obtained based on its original source (version 2015_09_01 in Table 2). GO in the UMLS has slightly more redundant is-a relations (1,576 v.s. 1,552) compared to the result obtained based on its original source of GO (version 2015_04_01 in Table 4). This difference may be caused by the a different version of GO (version 2015_04_04) integrated in the 2015AB release of the UMLS.

### Redundant is-a relations in randomly generated ontology

We have also tested FEDRR on randomly generated ontologies. There are two goals in testing FEDRR on randomly generated ontologies.

- To test the efficiency of FEDRR on arbitrarily large ontologies, and
- To compare the ratio of redundancy relations in real world biomedical ontologies with random ontologies with similar properties.

As each ontology can be represented as a DAG, we implemented Algorithm 2 to generate a random *D* *A* *G*(*N*,*E*,*C*_min_,*C*_max_), where *N* is the number of nodes, *E* is the number of edges, and *C*_min_/*C*_max_ are the minimum/maximum number of children a node can have.



Results of FEDRR on nine random ontologies are presented in Table 7. For each set of parameters, the median (using RR) of five runs is recorded. As can be seen from Table 7, FEDRR performs very well in terms of time efficiency and is suitable for online auditing of ontologies. It also scales very well to handle more than 1 million nodes and relations.

**Table 7:** Summary of the results for randomly generated ontologies

(*N*,*E*,*C*_min_,*C*_max_)	# Layers	TC	RR	RR%	T(ms)		
(500,000, 550,000, 2, 5)	12	5,957,690	6	0.0001	5,847		
(500,000, 550,000, 2, 10)	8	4,036,792	12	0.0003	5,212		
(500,000, 550,000, 2, 20)	6	3,000,751	7	0.0002	5,637		
(500,000, 600,000, 2, 20)	6	3,567,691	13	0.00036	5,449		
(500,000, 700,000, 2, 20)	6	4,190,494	34	0.00081	5,332		
(500,000, 900,000, 2, 20)	6	7,104,749	109	0.00153	8,183		
(500,000, 1,300,000, 2, 20)	6	25,934,499	1,404	0.0054	33,235		
(1,000,000, 1,200,000, 2, 20)	7	7,071,813	21	0.0003	18,051		
(1,000,000, 1,400,000, 2, 20)	7	8,694,703	133	0.0015	11,582		

*N*: number of concepts, *E*: number of is-a relations, *C*_min_/*C*_max_: minimum/maximum number of children a node can have, TC: number of transitive closure pairs, RR: number of redundant is-a relations, T(ms): time taken in milliseconds

The results also clearly indicates that the number of redundant relations increases when the density of the edges increases. For comparable density of edges, the ratio of redundant relations in SNOMED CT (US edition) (ranging from 0.0039 to 0.0058 %) and GO (ranging from 0.096 to 0.289 %) is much higher than in randomly generated ontologies (ranging from 0.0001 to 0.0054 %).

### Change rates in ontology evolution

Most ontologies in life science evolve continuously to account for new discoveries [11, 13, 14]. Our experimental studies indicated that the change rates of redundant is-a relations are significantly higher than the change rates of all is-a relations for both SNOMED CT and Gene Ontology.

**Fig. 4:**
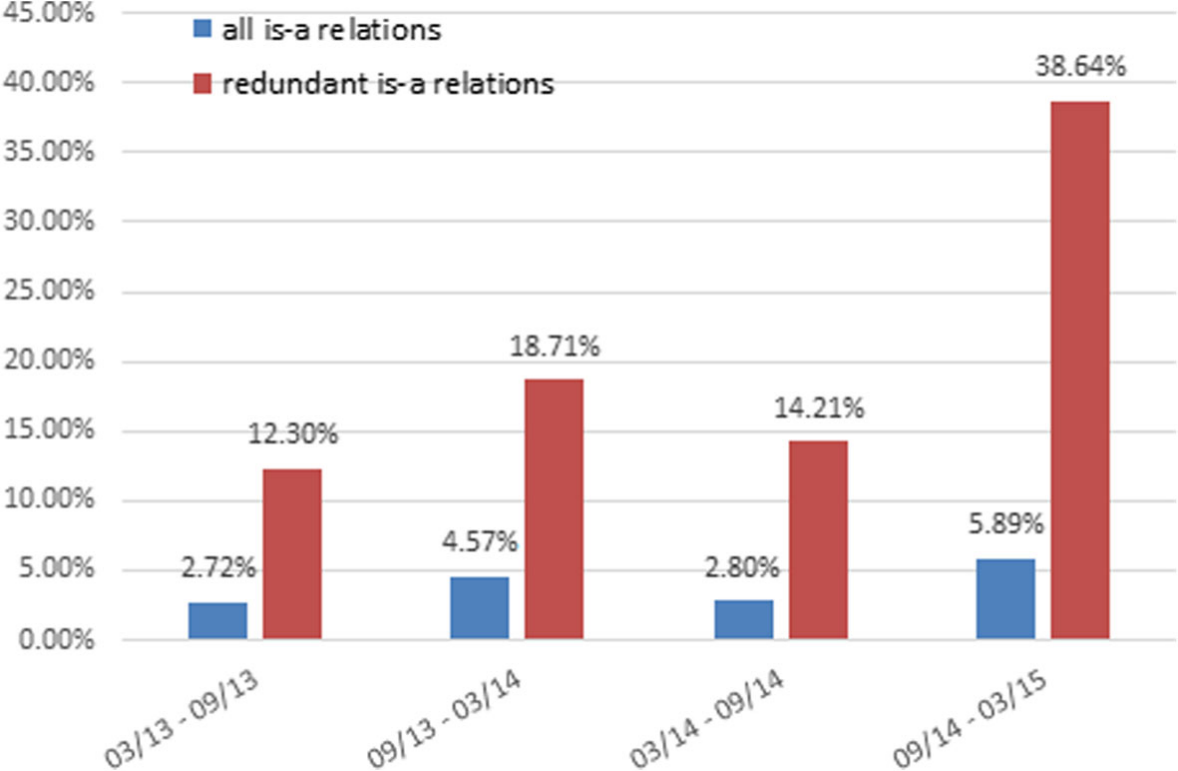
Change rate of redundant is-a relations compared to the change rate of all is-a relations during SNOMED CT evolution

**Fig. 5:**
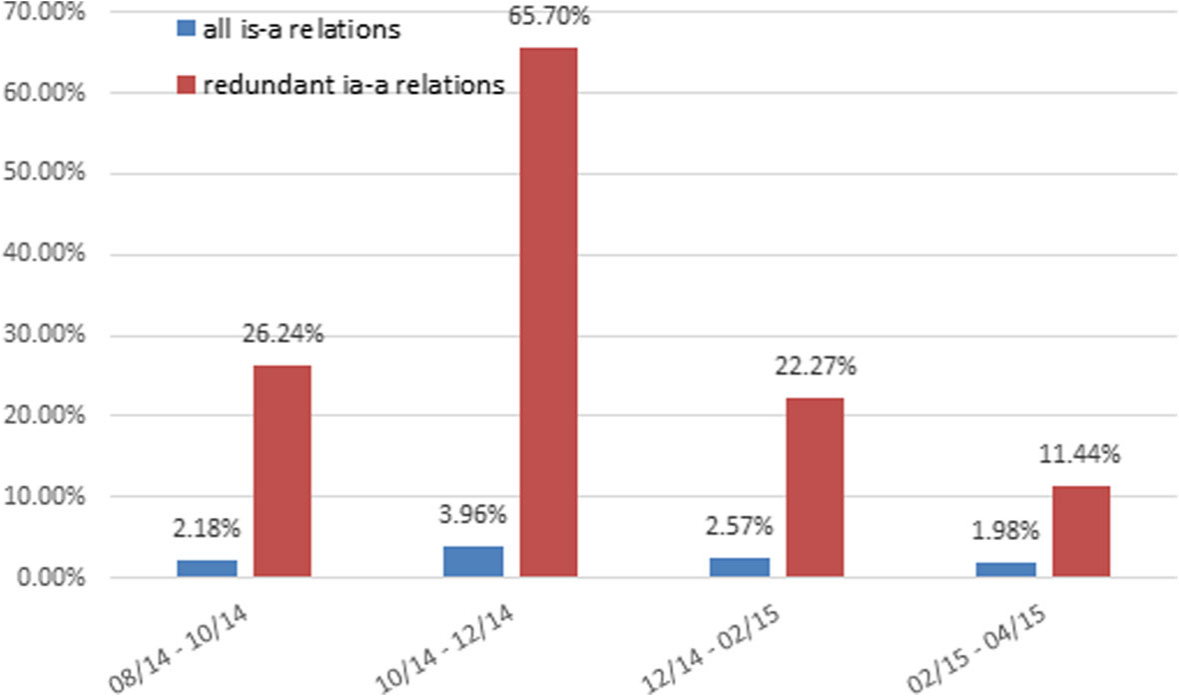
Change rate of redundant is-a relations compared to the change rate of all is-a relations during Gene Ontology evolution

Figure 4 is a graphical summary of the differences between SNOMED CT versions using the change rate of redundant is-a relations versus the change rate of all is-a relations. The change rate between two versions is defined as (|*N*−*O*|+|*O*−*N*|)/(|*O*|+|*N*|), where *N* is the set of relations in the new version, *O* is the the set of relations in the old version, *N*−*O* is the set of relations in the new version but not in the old version, and *O*−*N* is the set of relations in the old version but not in the new version. It should be noted that *N*−*O*≠*O*−*N* and |*N*−*O*|≠|*N*|−|*O*| in general. Figure 5 shows the summary of the differences between Gene Ontology versions using the change rate of redundant is-a relations versus the change rate of all is-a relations.

As can be seen from Figs. 4 and 5, the change rates of redundant is-a relations between versions are consistently higher than that of all is-a relations for both SNOMED CT and Gene Ontology. For SNOMED CT, the change rates of redundant is-a relations are 4 to 10 times higher than that of all is-a relations. For Gene Ontology, the change rates are 5 to 12 times higher. This indirectly validates the importance of eliminating redundant relations from ontologies during ontological evolution.

### Revision reversals of redundant relations

A particular case of ontological changes during ontology evolution is the revision reversal of relations, which refers to the addition (removal) of a relation being reversed by a removal (addition) in later versions. For example, the relation “Telangiectasia of skin of face (disorder)” *is-a* “Disorder of skin of head (disorder)” was not present in the 2013-09-01 version. It was added in the 2014-03-01 version, but was subsequently removed in the 2015-03-01 version. The following is a list of redundant is-a relations added in the 2014-03-01 version, but removed from the 2015-03-01 version.

1 $$ \begin{array}{lll} 131461000119105 & \quad\text{is}-\text{a~} &\quad 275544003 \\ 699056001 &\quad \text{is}-\text{a~} &\quad 400082007 \\ 699056001 & \quad\text{is}-\text{a~} &\quad 118930001 \\ 234140000 & \quad\text{is}-\text{a~} &\quad 400082007 \end{array}  $$

Clinically, such revision reversals may indicate the confusion about representing the relations among a group of concepts, which is closely related to the higher change ratio presented in Figs. 4 and 5. For SNOMED CT, we have observed much higher rate of revision reversals among the redundant is-a relations (19 out of 415; 4.58 %) compared to all is-a relations (1575 out of 491,825; 0.32 %). The revision reversal results in even higher rate when considering the segment induced by the redundant relation (59 out of 415; 14.22 %). For Gene Ontology, the rate of revision reversals among the redundant is-a relations (8 out of 1,614; 0.50 %) compared to all relations (188 out of 72,729; 0.26 %). The revision reversal results in even higher rate when considering the segment induced by the redundant relation (28 out of 1624 or 1.72 %).

### Evaluation

**Fig. 6:**
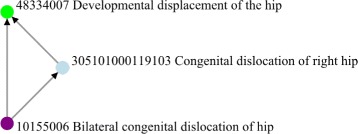
A visualized example of redundant is-a relation in SNOMED CT

Even though in most cases redundant edges should be removed, in some cases the redundancy is caused by a mistake of an edge along the indirect path. For example, in Fig. 6, the assertion that “Bilateral congenital dislocation of hip” is-a “Congenital dislocation of right hip” is most likely in error. This is because a concept involving “bilateral” should not be a subclass of a concept of limited laterality: “right” (but not “left”). Removing this edge would have automatically eliminated the redundancy of the detected relation.

**Fig. 7:**
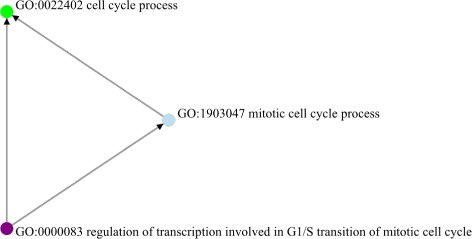
A visualized example of redundant is-a relation in GO

To evaluate the performance of FEDRR’s detection of redundant is-a relations from original sources of SNOMED CT and GO, a random sample of 30 redundant relations from SNOMED CT (2015-03-01 version) and 50 from GO (2015-05-01 version) were selected and manually reviewed by two human annotators. One annotator was asked to manually verify if the redundant hierarchical relations identified by FEDRR are correct. The other annotator was asked to review each redundant relation and provide on feedback if the redundant relation (direct edge) should be removed or an edge in the indirect path should be removed. For instance, Fig. 7 presents an example of redundant relation from GO manually reviewed by the second annotator. In this case, the annotator’s feedback is to remove the direct edge. For cases like the one shown in Fig. 6, the annotator’s feedback is to remove the indirect edge that is incorrect.

The first annotator verified that all of the redundant is-a relations identified by FEDRR are correct, that is, 100 % accurancy. Table 8 shows the feedback of the second annotator. Among 30 redundant is-a relations in SNOMED CT, 24 (80 %) should have direct edge removed, and 6 (20 %) should have indirect edge removed. Among 50 redundant is-a relations in GO, 45 (90 %) should have direct edge removed, and 5 (10 %) should have indirect edge removed.

**Table 8:** Numbers of direct edge and indirect edge that should be removed for 30 redundant is-a relations in SNOMED CT and 50 in Gene Ontology

	Remove direct edge	Remove indirect edge
SNOMED CT	24 (80 %)	6 (20 %)
Gene Ontology	45 (90 %)	5 (10 %)

To evaluate the performance of FEDRR’s detection of redundant hierarchical relations from UMLS, a random sample of 30 redundant relations detected from the SNOMEDCT_US in the UMLS was selected and manually reviewed by the first annotator. The annotator verified that all the 30 redundant relations identified by FEDRR are correct (100 % accuracy).

## Discussion

### Related work

There has been related work on exploring redundant relations in biomedical ontologies or terminologies [24–28]. Bodenreider [24] investigated the redundancy of hierarchical relations across biomedical terminologies in the UMLS. Different from Bodenreider’s work, FEDRR focuses on developing a fast and scalable approach to detect redundant hierarchical relations within a single ontology.

Gu et al. [25] investigated five categories of possibly incorrect relationship assignment including redundant relations in FMA. The redundant relations were detected based on the interplay between the *is_a* and other structural relationships (*part_of*, *tributary_of*, *branch_of*). A review of 20 samples from possible redundant part_of relations validated 14 errors, a 70 % correctness. FEDRR differs from this work in two ways. Firstly, FEDRR aims to provide an efficient algorithm to identify redundant hierarchical relations from large ontologies with 100 % accuracy. Secondly, FEDRR can be used for detecting redundant relations in all DAGs with the transitivity property.

Mougin [26] studied redundant relations as well as missing relations in GO. The identification of redundant relations was based on the combination of relationships including *is_a* and *is_a*, *is_a* and *part_of*, *part_of* and *part_of*, and *is_a* and *positively_regulates*. FEDRR’s main focus is to provide a generalizable and efficient approach to detecting redundant hierarchical relations in any ontology, which has been illustrated by applying it to all the UMLS source vocabularies. Moreover, the redundant hierarchical relations detected by FEDRR were evaluated by human experts, while only number of redundant relations was reported in [26] without human annotator’s validation.

Mougin et al. [27] exhaustively examined multiply-related concepts within the UMLS, where multiply-related concepts mean concepts associated through multiple relations. They explored whether such multiply-related concepts were inherited from source vocabularies or introduced by the UMLS integration. About three quarters of multiply-related concepts in the UMLS were found to be caused by the UMLS integration. Additionally, Gu et al. [28] studied questionable relationship triples in the UMLS following four cases: conflicting hierarchical relationships, redundant hierarchical relationships, mixed hierarchical/lateral relationships, and multiple lateral relationships. It was reported in [28] that many examples indicated that questionable triples arose from the UMLS integration process.

Bodenreider [29], Mougin and Bodenreider [30], and Halper et al. [31] studied various approaches to removing cyclic hierarchical relations in the UMLS. Although no cycles have been detected in the current UMLS in terms of the AUI, such approaches ([29–31]) to detecting and removing cyclic relations are needed before FEDRR can be applied. This is because FEDRR is based on the topological sorting of a graph, which requires no cycles in a graph.

### Future work

Although we have focused on investigating redundant *is-a* hierarchical relations in this paper, FEDRR is a general method and is applicable to detect redundant relations in other hierarchical structures. In future work, we plan to apply FEDRR on other hierarchical relationships such as *part_of* in SNOMED_CT and FMA, and *branch_of* in FMA.

## Conclusions

Detecting and removing redundant relations is an important quality improvement task for biomedical ontologies because non-redundancy is the basic premise of all semantic measures derived from ontological structures, such as semantic distance between concepts and ontology mapping and alignment. We introduced FEDRR for fast and exhaustive detection of redundant hierarchical relations in ontological hierarchies. Our algorithm runs in linear time to the size of the ontological structure in practice.

Using FEDRR, we performed systematic and exhaustive search of redundant is-a relations in two large ontological systems in biomedicine: SNOMED CT and Gene Ontology, as well as all the source vocabularies in the UMLS. The algorithmic core of FEDRR is easy to implement and extremely efficient. In our extensive experiments on real-world, large ontological structures, it took less than 20 seconds for FEDRR to process SNOMED CT and Gene Ontology. Moreover, FEDRR is a general approach and can be applied to detect redundant relations in other hierarchical structures.

With these results, we believe that FEDRR is production ready. It is available at https://github.com/gmingx/fedrr.

## Abbreviations

AUI: Unique atom identifier
CUI: Unique concept identifier
CWA: Closed-world assumption
DAG: Directed acyclic graph FEDRR: Fast, exhaustive detection of redundant relations
FMA: Foundational model of anatomy
GO: Gene ontology
HPO: Human phenotype ontology
NLM: National library of medicine
NCI: NCI thesaurus
OQA: Ontology quality assurance
OWA: Open-world assumption
SNOMEDCT_US: SNOMED CT US edition
SCTSPA: SNOMED CT Spanish language edition
UMLS: Unified medical language system
